# Communicating With Parents and Preschool Children: A Qualitative Exploration of Dental Professional-Parent-Child Interactions During Paediatric Dental Consultations to Prevent Early Childhood Caries

**DOI:** 10.3389/fpubh.2021.669395

**Published:** 2021-05-12

**Authors:** Siyang Yuan, Gerry Humphris, Lorna M. D. MacPherson, Alistair L. Ross, Ruth Freeman

**Affiliations:** ^1^Dental Health Services Research Unit, University of Dundee, Dundee, United Kingdom; ^2^Health Psychology, School of Medicine, University of St Andrews, St Andrews, United Kingdom; ^3^School of Medicine, Dentistry & Nursing, University of Glasgow, Glasgow, United Kingdom

**Keywords:** communication, conversational analysis approach, paedodontics, dental professional, parent, pre-school child, utterances, cues

## Abstract

The aim of this study was to explore communication interactions and identify phases adopted by dental professionals with parents and their young children and to examine the hypothesis that successful social talking between the actors together with the containment of worries allows the formation of a triadic treatment alliance, which leads to achieving preventive dental treatment goals. Conversation analysis of the transcribed data from video recordings of dental professionals, parents and preschool children when attending for preventive dental care was conducted. The transcriptions were read, examined and analysed independently to ensure the trustworthiness of the analysis. The transcriptions were explored for interactive patterns and sequences of interaction. Forty-four individual consultations between dental professionals, parents, and preschool children were recorded. The number of communication behaviours was 7,299, with appointment length ranging from 2 min 10 s to 29 min 18 s. Two patterns of communication were identified as dyadic (between two people) and triadic (between three people) interactions within a continuous shifting cycle. The three phases of communication were social talking, containing worries and task-focusing. Social talking was characterised by shifts between dyadic and triadic communication interactions and a symmetry of communication turns and containing worries. This typified the cyclical nature of the triadic and dyadic communication interactions, the adoption of talk-turn pairs, and triadic treatment alliance formation. Task-focusing pattern and structure were different for dentists and extended-duty dental nurses. For dentists, task-focusing was characterised by a dyadic interaction and as an asymmetrical communication pattern: for extended-duty dental nurses, task-focusing was typified by symmetrical and asymmetrical communication patterns within dyadic and triadic interactions. Empathy and understanding of the young child's emotional needs during containing worries allowed the formation of the triadic treatment alliance and with this treatment alliance, the acceptance of interventions to prevent early childhood caries during “task-focusing.” This qualitative exploration suggests that dyadic and triadic communication interactions are of a dynamic and cyclical quality and were exhibited during paediatric dental consultations. The communication phases of social talking, containing worries and task-focusing were evident. Successful social talking signalled the entry to containing worries and triadic treatment alliance formation which permitted the preventive goals of the consultation to be achieved (task-focusing). Future work should generate additional data to support the hypotheses created here namely that, social talking and containing worries triggers an integral pathway to task-focusing and the achievement of preventive dental goals.

## Introduction

The United Nations Convention on the Rights of the Child (UNCRC) Article 12 secured children's rights and influenced policies for children to be included and to be heard in their healthcare (1, 2). Children aged as young as 4 years of age, when accessing health services with their caregivers, are said to be able to recall information, would like more involvement in the discussions concerning their health and wish to have a say about their treatment (3, 4). Some evidence suggests that young children can engage with the health professional, although their participation in the consultation is limited (5). Nonetheless, children do experience barriers in their understanding and voicing their opinions (6). The proposed asymmetrical interaction between caregivers and the health professional contributes to a dilemma for the health professional when attempting to engage with the child patient in active treatment (7). The paediatric dental appointment is no different.

The paediatric dental consultation, in addition to the above, is fraught with worries and concerns. For the dental professional, the interaction with parents and children may be troublesome because of the relationship between parental and child dental anxiety and the wish, on the part of the dental professional, to maintain a two-person interaction with the parent, unwittingly, at the expense of the child (8, 9). For the parent there are worries that the child will accept treatment and will be able to manage the encounter with the dental professional. For the child all is strange and unfamiliar. It is in this setting that dental professionals must provide dental care using their communication and behavioural management skills to reduce the dental anxieties and other parental and child concerns. Effective communication is, thus, essential for successful dental care outcomes in the paediatric clinic.

To achieve this goal, dental professionals must ensure that both the parent and the child are involved and have the opportunity to contribute and speak during the treatment appointment. This is an important step since effective communication strategies will contain implicit or explicit parental and child dental anxieties and permit through sensitive enquiries the formation of a special type of treatment alliance. This treatment alliance is different from that between the adult patient and dental professional which may be thought of as an adult-to-adult interaction in which the patient accepts the treatment the health professional is offering. The dental professional, however, when caring for children must form the treatment alliance with the child through the parent (9). This treatment alliance may be referred to as a triadic treatment alliance and the interaction, within the alliance, categorised as triadic communication between health professional, parent and child (10, 11). The dental professional by promoting and maintaining this three-way conversation upholds the treatment alliance with the child that can enable successful treatment outcomes. However, with the pre-school child, communication is complex. This is strongly related to the stage of the child's cognitive and emotional development. Therefore, the dental professional needs first, to establish rapport with both parent and child, secondly, engage in information gathering and respond to questions from the parent and thirdly, acknowledge the complexity of the verbal and non-verbal exchanges between the three “actors” (i.e., the dental professionals, parents and the young children). Intrinsic, thus, to the treatment alliance is effective verbal and non-verbal communication together with the containment of patient worries and concerns by the health professional.

In addition, health professionals must focus on the task at hand and exchange information with the parent to ensure that all clinical safeguards are maintained (6). This is echoed in Kelly et al.'s work with a warning, that “when the emphasis moves to parents as consumers of paediatric healthcare, children are at risk of being objectified or even marginalised” (12). Empirical work, has suggested, that when dental nurses are trained in communication skills, they can provide effective oral health interventions for and with the young child and their caregiver (13). More recent research supports this finding. It has revealed that when caregivers/parents are actively involved in their child's dental health care, their interventions promote the transfer of knowledge (14). Nevertheless, when parents unwittingly become an “interpreter” for their child, translating the dental professionals' words into a clear, understandable form, there is the danger that parental utterances may hinder rather than enable child patient-centred care (15, 16). It is known that when children are unintentionally excluded from the interaction between practitioner and parent, they interrupt and interject with talk and gestures. Jenkins et al. (17) describe these encounters as “instigating talk” to attract the attention of the parent to contribute to the conversation. This demonstrates a number of interactive processes, such as for example social talk, that occur during the appointment with the child patient. Following Tannen's formulation, this talk may be conceptualised as “communication scripts” which Tannen proposes are important for the different phases of the paediatric appointment between professional, parent and child. The significance of these different communication scripts we suggest, is that they enable the professional to respond and to assist the child and parent to navigate from examination to treatment. Our previous work (18), limited to the immediate effects of the dental professional's words on the child's behaviour, would support this interpretation of Tannen's thesis (19). However, what remains unclear, is the relevance of such communication interactions and phases and how they affect children's engagement and acceptance of treatment in the dental setting. While we acknowledge the salient work of Bridges et al. (16) and Wong et al. (14) we believe that it is the nuances of the communication interactions and subsequent phases that are of central importance. The aim of this study, therefore, was to explore communication interactions and identify phases adopted by dental professionals with parents and their young children and to examine the hypothesis that successful social talking between the actors together with the containment of worries allows the formation of the triadic treatment alliance, which leads to achieving preventive dental treatment goals.

## Methods

### Study Design

The present study is an analysis of the video recordings from the BEHAVE2 study (18, 20). The BEHAVE 2 study, video recorded 44 individual paediatric dental appointments between dental professionals, preschool child patients and their parents. In total the number of communication verbal and non-verbal behaviours recorded was 7,299. All turns had been given a unique behavioural code in the original primary analysis. The rate of all communication instances was: 5.92 instances/min for the dental professional; for the parent 3.22 instances/min and for the child 1.06 instances/min. The length of the appointments ranged from 2 min 10 s to 29 min 18 s. We adopted a qualitative exploration of the transcriptions of the video recordings and used a conversation analytic approach to scrutinise the data.

### Setting

The Childsmile Programme is funded by the Scottish Government, whose purpose is to reduce child dental health inequalities using the proportionate universalism approach (21). As part of the programme, parents of children aged as young as 2 years, are encouraged to access primary dental care for fluoride varnish applications twice a year to prevent early childhood caries. During such appointments, the dentist or the Extended Duty Dental Nurse (EDDN) will apply fluoride varnish to the child and discuss toothbrushing regimes and healthier diets with the parent/caregiver. Since the type of treatment as well as the age of the child affects the relationship between parental and child dental anxiety (8), the relative non-invasive nature of the Childsmile appointment provides a perfect occasion to examine communication processes in the paediatric dental appointment.

### Participants

Purposive sampling was used to identify dental professionals who participated in the Childsmile programme in general dental practises located in East of Scotland. Four general dental practises were approached and agreed to take part, which included urban and rural practises in affluent and deprived areas. Five dental professionals working in these practises agreed to participate in the study and completed the written consent form. Fifty child-parent dyads were approached and invited to take part with the following six pairs being excluded due to: (i) two pairs of twins were treated with their twin siblings; (ii) one child was the sibling of the participating child and was invited by the parent to receive fluoride varnish application during the video observation; (iii) one child was excluded due to observed learning difficulties; and (iv) two children declined to take part.

### Data Collection

Paediatric dental consultations were video recorded to capture both the verbal and non-verbal communication. Each consultation included, toothbrushing with fluoride toothpaste and dietary advice, and a fluoride varnish application. All the video recordings were collected during May – September 2017 (20).

### Data Analysis

Conversation analysis was used to analyse the transcribed video data (22–24). The conversation analysis approach is particularly well-suited to video recordings within real-life scenarios of clinical interactions where wide variation of both content and participation of “actors” is apparent. Bridges et al. (16) suggest that conversational analysis is an appropriate form of qualitative analysis in the dental setting since it permits “the specific qualities of patient-centred care” to be realised and the “sequential patterns of activity” during the dental visit to be identified. High definition quality video clips of a series of appointments enabled detailed transcripts of the verbal and non-verbal content and behaviour to be prepared as the data corpus for members of the research team to apply conversational analysis.

The transcriptions were investigated for interactive patterns and sequences of interaction. The analytical purpose was to examine how the “actors” interacted, rather than an in-depth examination to explain choice of communication behaviours (22). Therefore, in this instance conversation analysis focused on the “talk-in-interaction” in the dental setting. Conversation analysis was used to capture the details of the turns taken in the conversation in terms of the timing, the subtleties of the utterances between the speakers including the phrasing, the patterns of stress, the intonation as well as non-verbal behaviours.

In this analysis, for each paediatric dental consultation, the sequential structure, turn-taking, and patterns of turns of the communication were analysed using Finset and Ørnes' (25) theoretical understanding of clinical encounters. Garrod and Pickering outline a helpful framework to understand some of the subtle phenomena that can be identified in many clinical interactions. They highlight patterns of interactions within the consultation that go very smoothly and “speakers apply largely automatic and unconscious processes of interactive alignment in the process of speaking and listening in conversations” (26). Communication in these instances according to Finset and Ørnes “becomes more symmetrical and with a higher degree of mutuality” (25). These patterns are identified from close attention to the individual turns taken by the actors. Many of these turns are linked to exhibit these key phenomena within consultations. For clarity, we adopted the definition of turn-taking as, “the single speech turn (i.e., continuous speech by [*the actors*] that is preceded and followed by the other's speech) can therefore contain more than one utterance” (27).

The phase of the communication was informed by the hypothetical model of clinician-patient interaction of Szasz and Hollender (28). In their theoretical paper Szasz and Hollender proposed three different models of the clinician-patient interaction (Table 1). The first of these is the activity-passivity model in which the health professional does something to the patient and the patient receives and accepts the care provided. The activity-passivity model reflects the paternalistic model of the dentist-patient interaction in which the dentist is active and the patient passive (29). The second model is guidance-cooperation. Within this interaction the health professional tells the patient what to do and the patient obeys accordingly. The guidance-cooperation model is evocative of the dental check-up visit in which to quote Coleman and Burton (30) “the patient knows something; dentist knows something.” Therefore, there is joint knowledge in the guidance-cooperation model, and while the health professional listens to the patient, it is the health professional who makes the final treatment decision. The last part is mutual-participation model in which the health professional and patient are joint partners. This is distinctive since the patient is active in the choices and decisions with the health professional regarding their health care (28). Hence, we applied in parallel through conversation analysis the close examination of turns to identify features of various aspects of interactive alignment across possible phases of the consultation as described by Szasz and Hollender.

**Table 1:** The three basic models of the health professional-patient interaction of Szasz and Hollender (28).

**Model**	**Health professional role**	**Patient role**	**Application**	**Prototype of the model**
Activity passivity	Does something to the patient	Accepts and receives the treatment	Treatment	Parent to child
Guidance cooperation	Listens to the patient; tells the patient what to do; makes the treatment decisions	Speaks with the health professional but accepts the treatment decisions	Examination appointment	Parent to child
Mutual participation	Advises and negotiates treatment decisions	Patient in equal partner care	Negotiation of treatment or preventive plans	Adult to adult

The process of conversation analysis, adopted here, included (31):

1. Selecting relevant interactions

During the initial viewing of the video data interesting moments or “noticings” relevant to the research question were logged by SY and RF, separately. These are also referred to as “connexions” by Finset and Ørne (25). This was a slow and arduous process as the videos were examined frame-by-frame. Following, this first tranche of the video data, the identified incidents were watched, and the process repeated. During this time SY and RF watched, shared and discussed the choices made (31). Finally, the incidents or “episodes” transcribed were those which focused on the question under investigation. Viewing the videos frame-by-frame permitted the video data in the form of “stills” (e.g., positioning of the child and parent during the appointment) to supplement the transcribed data.

2. Identifying recurrent interactional patterns

SY and RF returned independently to examine the transcribed episodes in more detail with regard to how the EDDNs and parents engage the child in the interaction; how the child responded in turn to any invitation to speak by EDDN or parent, and the triadic communication patterns identified from the turn-by-turn analysis.

3. Analysing the excerpts on the micro-level

The specifics of the conversation including the following: turn-taking, the sequence of turns, interruptions and pauses, tone of voice, pitch of speech, selection of words were also analysed to examine the communication phases and conversational strategies that each speaker used in the health encounter. Non-verbal behaviours such as gaze and positioning were also examined from the supplementary video material. Positioning, for example, was noted if the child was sitting on the parent's lap, or sat adjacent or opposite to the parent.

4. Trustworthiness of the data analysis

The transcriptions were read, examined carefully and analysed independently by SY, RF and GH to ensure the trustworthiness of the data analysis. They examined the transcripts to identity sequence, turn-taking etc. from the thick descriptions of the data. When a difference occurred, this was discussed between SY and RF. In the instance where consensus could not be reached, GH was asked to contribute thus ensuring consensus and achieving confirmability. Using their clinical and social knowledge of paediatric dentistry, permitted SY and RF to have a cogent understanding of utterances during the consultation; SY (20) and GH (32) have in-depth knowledge of video analysis of communication and RF is experienced in qualitative methodologies. In view of this expertise the credibility of the data analysis was ensured.

5. Presentation of the transcriptions

We have taken into consideration, the complex transcription symbols that are used in conversational analysis when presenting verbatim transcripts. To enhance the readability and better understanding of the selected excerpts, we have simplified the detailed transcription symbols (15) in the verbatim transcripts (Table 2).

**Table 2:** Conversational analytic transcription symbols (16).

**Symbol**	**Description**
[ ]	Overlapping speech
↑	Upward shift in pitch
↓	Downward shift in pitch
Wor:d	(Colon) Prolongation of sound
word	(Underline) Emphasis
WORD	(CAPITALISED word) Section of talk that is relatively loud than the surrounding talk
°word°	(Degree mark) Section of talk that is relatively quieter than the surrounding talk
(( ))	Transcriber's comments including non-verbal behaviours
=	No gap between the two turns.
X:X	An underlined colon within a syllable indicates that the intonation within the syllable falls then rises.
XX:	An underlined second letter within a syllable followed by a non-underlined colon indicates that the intonation within the syllable rises then falls.

6. Anonymity and confidentiality

All of the children's names provided in the extracts presented have been changed to ensure the anonymity of the participating child and parent.

## Results

Forty-four paediatric dental consultations were video recorded. The children were aged between 24 and 70 months, 21 were boys. The participating dental professionals had varied experience with one in their first year since qualification, and the remaining having between 5 and 10 years clinical experience. All accompanying caregivers were mothers, except on five occasions where a grandparent (*n* = 1) and a father (*n* = 4) accompanied the children. Please note that the generic term parent will be used to describe all caregivers.

### Recurrent Patterns and Communication Phases

The results are presented in the order of the dental consultation, starting with the dental professional welcoming parent and child, information gathering, answering questions and moving to the objective of the Childsmile visit, that is to provide oral health education and fluoride varnish application. The extracts provide examples from the transcriptions all of which showed features of the communication patterns and communication phases described below.

Two recurring patterns of communication were identified within the duration of the appointment and were dynamic in their nature. We propose that two communication interactions primarily occurred during the Childsmile appointments explored here: the first a dyadic dental professional-parent, and/or dental professional-child and/or parent-child interaction and secondly a triadic dental professional-parent-child interaction which emerged as a continuous shifting cycle of dyadic and triadic interactions. Three phases of communication interaction were observed. These were (i) social talking, (ii) containing worries and (iii) task-focusing. To illustrate in greater detail the three communication phases, key excerpts are presented below and in order.

### Communication Phase 1: Social Talking

The following two extracts are illustrative of shifts in social talking to form or maintain triadic communication interactions. In the first extract, Jack a 4-year-old knows the dental professional (EDDN) well: in the second extract Mike a 5-year-old is visiting the practise for the first time. It is evident that the communication interactions used by the same dental professional in the two scenarios provided was different.

In the first extract (Extract 1), the dental professional's social talking is more direct to both Jack and mother and the following discussion is between all three participants. In the second, Mike is in a new setting, he is hesitant and relies on mother to speak first and support him in his engagement with the dental professional's social talking. This is reflected in the number of utterances in each extract. Jack has eight compared with five utterances from his mother and the dental professional: Mike and his mother have seven utterances each, while the dental professional has 13. This suggests that a symmetry in the triadic communication interaction existed between Jack, mother and the dental professional. This is demonstrated in the first few moments of the appointment - the questioning (DP: line 1), the support from mother (line 2) and Jack's answers about his sore knee and elbow (for example line 3, “I fell over today”) directly to the dental professional (20). The observed symmetry of the triadic communication interaction also reflected the guidance-cooperation (lines 1–3) and the mutual-participation (lines 4–19) phases of the relationship between parent, child and dental professional. The interaction between Jack and the dental professional echoed a lexical alignment with the same words being used between the adjacent pairs, for example in lines 3 and 4. The equality of the interaction, observed in the partnership working, were apparent and appeared to be characteristic of social talking within triadic communication.

**EXTRACT 1:** Jack returning to the practice.

1. DP: So [Jack], tell me, what have you been doing to your knee? ((when Mum put the boy onto her lap sitting in the tub chair opposite the dental chair))
2. Mother: ^°^What happened to your knee?^°^ ((when DP pointed to his knee with a plaster))
3. Jack: (He looked at his knee, then looked to the DP) I fell over today.
4. DP: TO:DAY↓= ((DP looked surprised))
5. Jack: =[Yeah] I did it last week ((showing his elbow to the DP, and then DP touched his wound on the elbow))
6. DP: Okay.
7. Jack: I did this to-day ((Boy's hands put on his knee)).
8. DP: It's a new one↓
9. Jack: Yeah ((Mum nodded and looked at the boy))
10. DP: What did you do? (0.4) You fell over? Where did you fall?
11. Jack: On…on the pavement.
12. DP: Haha haha…at school or…?
13. Mother: Haha…. [Yeah] nursery (0.8He's very literal. And he could be literal (and that could be funny). ((Both Mum and DP giggled))
14. M: Do you know that? ((Mum faced toward the child))
15. Jack: We were=
16. Mother:=[In] the nursery, wasn't it? ((Mum faced the child and waited for him to confirm))
17. Jack: Yeah…
18. Mother: Outside in the garden, wasn't it?
19. Jack: Yeah…

In contrast, the communication interaction between the dental professional, the parent and Mike was different and was typified by the dynamic quality of their communication interaction. During the opening part of the consultation, the interaction was observed as dyadic (lines 1–4) whereas for most of the interaction it was triadic (lines 7–35). As the shifts in dyadic and triadic communication were noted, a change was also observed in the symmetry of the turns, within the interactions, between Mike, parent and the dental professional. Adopting Finset and Ørnes' (25) theoretical model, we propose that the dental professional used her social talking (lines 1–10) to “shape [*Mike and mother's*] responses” (18), suggesting a “guidance-cooperation” phase of their interaction (Mike obeyed when told by the dental professional not to sit on the “tub chair,” line 3). Only after mother's comment of her child's shyness did the shift in communication phase become evident and move to a “more affiliated and facilitative communication” as noted in the change to a more symmetrical form (25). The dental professional's social talking appeared to be empathetic as evidenced by Mike's engagement (line 13). Therefore, in this instance, the dental professional's awareness and understanding of the child's shyness and interest in the cartoons on the surgery wall, enabled her to use the cartoon characters as a foundation of her social talking to Mike and mother. However, the closing down of mother's utterance, “Where's the shark?” by the dental professional by asking Mike, “Do you know why you are here today?”, suggested something different (lines 31–35). This sudden shift in topic implied that engagement between the three “actors” was interrupted. This interruption signalled a change from a symmetrical to an asymmetrical pattern within the triadic communication interaction, together with a shift from a mutual-participation (lines 7–31) to a guidance-cooperation (line 31–35) phase. The dental professional acted to “optimise” Mike and mother's responses to permit the goal of the dental appointment to be achieved, namely to show mother and Mike how to “Clean teeth↓” (line 35).

**Figure 1:**
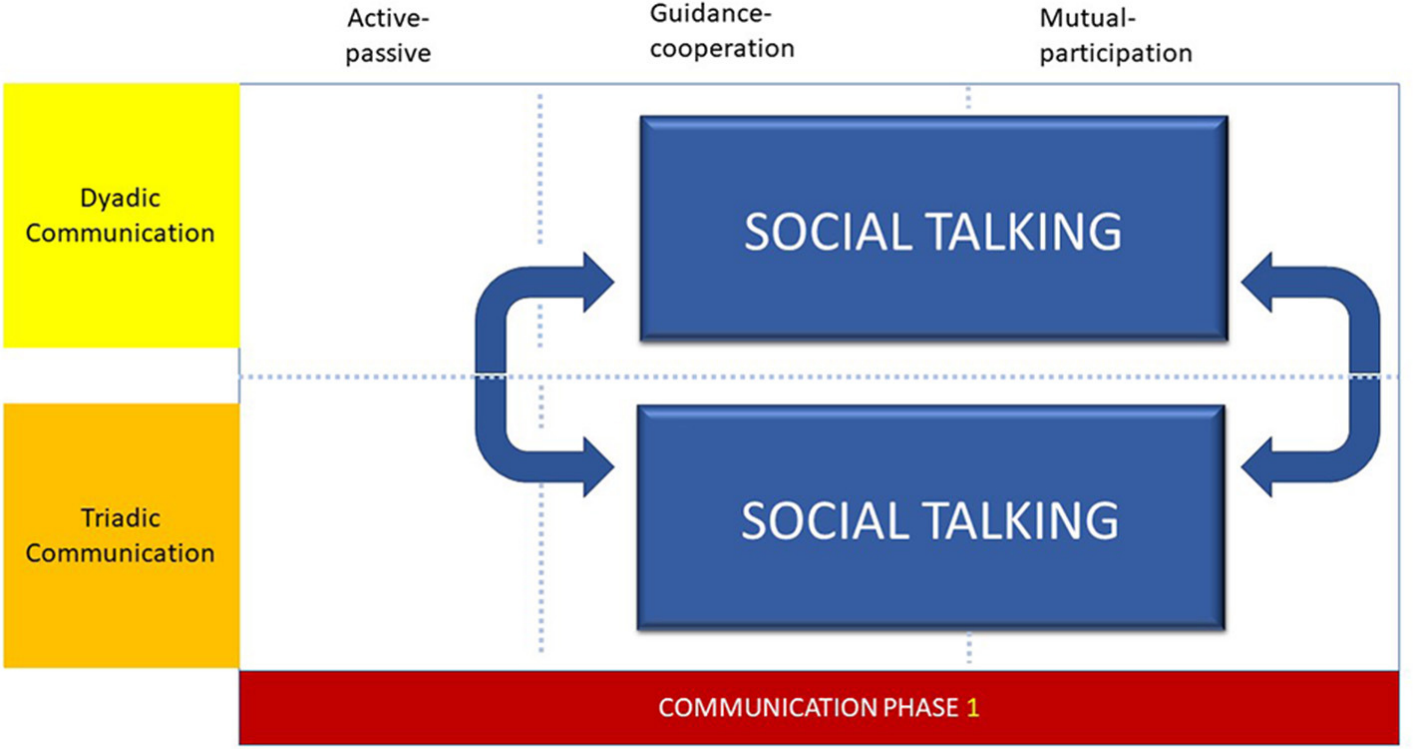
Communication phase 1.

Social talking (Figure 1) in the form of greeting and welcoming the parent and child, was used by all the dental professionals to speak and engage with parent and the child during the initial phase of the appointment. However, the length of time used for social talking was related to the type of dental professional. EDDNs spent longer and tended to use social talking to develop and maintain rapport with children and parents more readily than dentists (20). The success of social talking was also dependent upon the age and rapport building during previous appointments. Therefore, when the child was younger or not known to the practise, social talking was altered and adjusted to form a triadic communication interaction. Therefore, the social talking communication phase was characterised by shifts between dyadic and triadic communication interactions, in which contemporaneous changes are observed within the degree of symmetry of turns, that are suggestive of both guidance-cooperation and mutual-participation phases within the interaction.

### Communication Phase 2: Containing Worries

In Extract 2, the dynamic nature of the communication interaction is illustrated. This example shows the sequence of communication interactions exhibited by the participating dental professionals during their paediatric encounters with younger child patients. A careful exploration of the data suggests that a continuous shifting cycle of dyadic and triadic interactions assisted the dental professional to form a treatment alliance with the child via the parent, to achieve the goal of the appointment. Therefore, the interaction throughout this example was characterised by the communication phase “containing worries.” This phase of interaction contained all the elements of mutual-participation between all three “actors.” From the first moments of the meeting the dental professional aligned herself with Cathy, aged 4. This allowed Cathy's mother to observe the empathy expressed in the dental professional's utterance to Cathy as shown in lines 5–6. Allying herself with Cathy (lines 9 and 11) and using Cathy's own word “yuck,” the dental professional enabled further engagement with Cathy through mother (line 10). During the triadic interaction, both verbal and non-verbal cues (line 12) were used by mother to support the goal of the Childsmile appointment. Therefore, as the dental professional spoke of fluoride varnish, mother first verbally emphasised Cathy's reward and secondly playfully nudged her daughter's shoulder with her own by way of expressing the importance of Cathy's reward (line 12). It may be suggested that Cathy's laughing, smiling and “high-five” (line 14–15) reflected the containment of Cathy's worries and the formation of the treatment alliance.

**EXTRACT 2:** Cathy containing anxieties and worries and forming the triadic treatment alliance.

1. DP: So what we gonna do today↓ Do you know↑
2. Cathy: emm.em…((child shook her head, smiling at the nurse))
3. DP: Did they tell you↓ ((smiled at the child))
4. Cathy: Emm…em.((child nodded))
5. DP: Okay↓so… we gonna go over toothbrushing, we gonna talk about healthy eating, we gonna talk about sugary treats…We will play a game…
6. M: yea↓
7. Cathy: Huh↑((Child laughed))
8. DP: We will paint your teeth with this special paste like last time, and then we will give you a goody bag↓ ((nurse counted fingers to identify the number of agenda items))
9. Cathy: = =I don't like yucky banana toothpaste ((child put fingers on her nose showing dislike of the FV taste))
10. M: That's what you would say, haha.
11. DP: Yea…((nurse showing a yucky face to the child)) (2.0). But don't worry, we gonna SANDWICH it in beside good stuff ((showing a sandwich with two hands)). So↓ a game is good, paint's yuck, goody bag is GOOD ((nurse used hand gestures and nodding toward the child)).
12. M: Goody bag at the end, hhh… ((Mum used shoulder playfully to nudge the child))
13. DP: Yea↑ ((nurse nodded when looking at the child))
14. Cathy: ((Child laughed and showed a happy face))
15. DP: High fives ((Nurse reached her hand toward the child and then had a high-five with the child)). YEAH↓ So let's get the show on the road↓

The relationship between parental and child dental anxiety, anticipatory worries and fears of separation (9) are known to distort and influence the parent and child fully engaging in the dental appointment. In these situations, the caring dimension of dental treatment is misunderstood and perceived as frightening and to be avoided at all costs by the child. This is irrespective of the degree of invasiveness of the dental procedure (33). Therefore, the child attending for a fluoride varnish application may be as anxious about this non-invasive treatment as a child attending for an extraction. The awareness of the dental professional to identify and to acknowledge the child's treatment worries is of central importance. It is the dental professional's awareness to appreciate the child's emotional reactions, to identify the affect and to respond appropriately, that allows the parent to enter the encounter and achieve the formation of a treatment alliance with the child.

To contain worries the dental professional adopted the “adjacent talk-turn pairs” approach, with the parent and then the child. Providing information to the parent, the dental professional and parent work in unison to reduce child worries to form a treatment alliance. Once more the flow of these exchanges may be observed as shifts from triadic, to dyadic and back to triadic communication interactions. We proposed that it is the dental professional's empathy and understanding of the young child's emotional needs that allowed the formation of the treatment alliance and the acceptance of the preventive Childsmile treatment.

With the parent as an interpreter and decoder of information (34), the parent acted as a go-between, across dental professional and child. It is the parent who enables the child to receive the treatment being offered. In the following short extract, Bobby's superhero was Spiderman. Father used Spiderman to decode the dental professional's words to support Bobby and to understand why he should have the fluoride application:

“The dental professional described the fluoride varnish process to Bobby and father. Bobby looked tearful, ‘I don't want it!’ Father smiled and winked at Bobby. Father spoke of Spiderman's need for strong teeth and how the varnish would make Bobby's teeth as strong as Spiderman's. ‘Spiderman Bobby’ could do anything and with the varnish would have strong teeth like Spiderman.”

**Figure 2:**
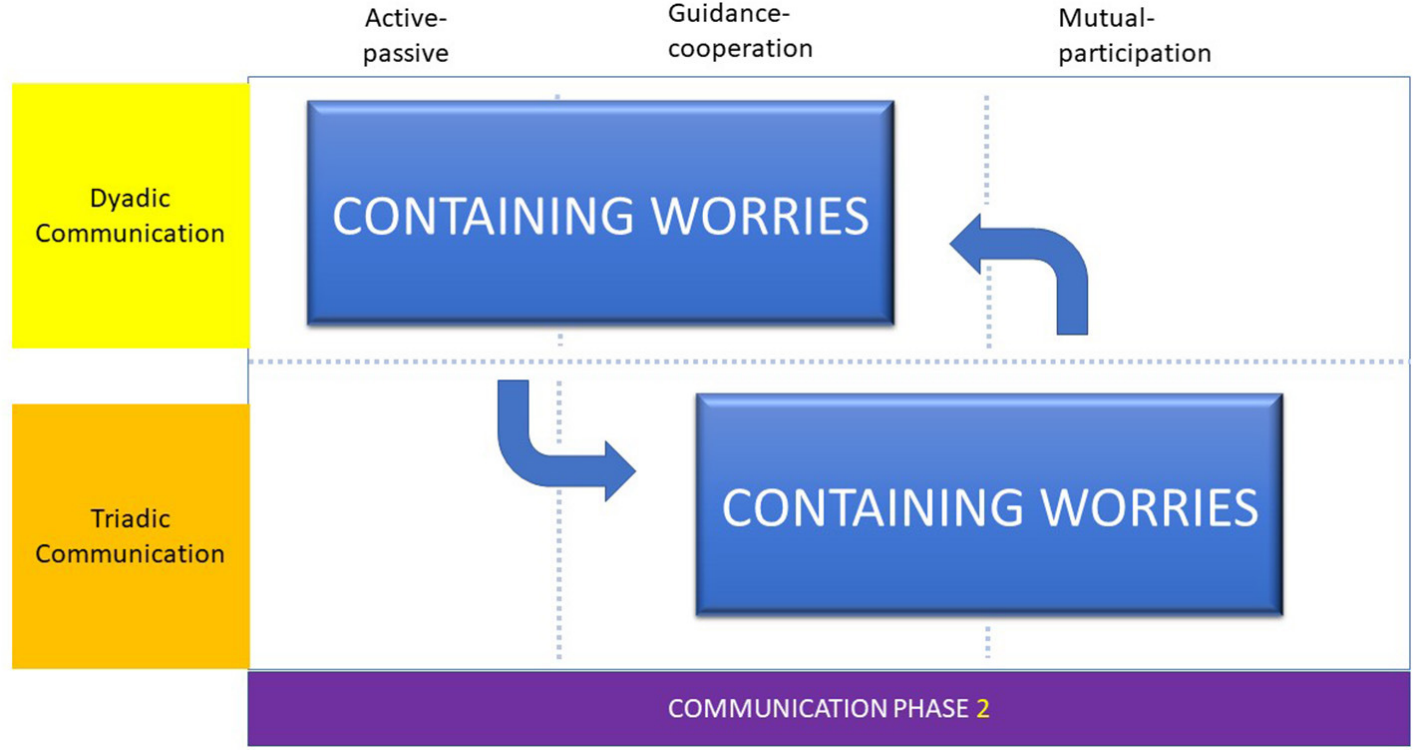
Communication phase 2.

Therefore, the communication phase, containing worries was typified by the cyclical nature of the triadic and dyadic communication interactions, the adoption of an “adjacent talk-turn pairs” approach, providing and gathering information and forming a treatment alliance (Figure 2).

### Communication Phase 3: Task-Focusing

Extract 3, shows the elements of task-focusing as the dentist speaks to a parent about the child's toothbrushing. Of particular noteworthiness was the predominance of the dyadic communication interaction between dentist and parent.

**EXTRACT 3:** Dentist asking mother about her child's toothbrushing.

1. D: And brush twice a day↑
2. M: Yes=
3. D: Yea↑ And you still help them↑
4. M: Yes=
5. D: =With both of them?
6. M: Yea

In comparison, Extract 4 shows an equivalent situation between EDDN, parent and Jane aged 3. In the encounter a different form of task-focusing was used. In this example, the task was to discover when the child brushed her teeth and although the character of the question-answer turn-taking is similar, Jane was now at the centre of the conversation from the start of the interaction (line 3). The number of utterances shows an asymmetrical communication pattern since the EDDN has only two utterances while mother and Jane both have seven. However, this extract illustrates the dynamic nature of communication with a symmetrical triadic communication interaction (lines 1–7) paving the way for a dyadic communication interaction between mother and child (lines 8–17). In this instance, the EDDN achieved the task of discovering the child's toothbrushing regime with mother's assistance. It may be proposed that two processes are in operation here. First, the mother acted as a translator for the EDDNs' questions and portions the EDDN's questions into understandable “chunks” and in doing so optimised Jane's responses (lines 11–17). The second process belongs with the EDDN. In terms of “adjacent talk-turns,” the EDDN provided expressions for mother to use to translate and thereby enabled Jane to speak, provide answers and for the task to be achieved. In subsequent discussions between EDDN, parent and Jane, the triadic communication interaction was restored, and a symmetrical pattern re-established.

**EXTRACT 4:** EDDN asking mother and Jane about toothbrushing.

1. EDDN: Does a grown-up still help you brush your teeth?
2. M: (2.0) Who helps you a lot?
3. Jane: Hmm…
4. EDDN: Who helps you?
5. Jane: Mummy
6. M: Uh:huh:
7. EDDN: Good↓ And how many times a day do you brush your teeth?
8. Jane: Emm…
9. M: Can you think about the best answer?
10. Jane: Hmm….(then gaze at Mum)
11. M: When do you do it? (1.0) Do you do it in the morning…before = =…nursery~
12. Jane: Yeah (nodded)
13. M: And then…once before::
14. Jane: Bedtime.
15. M: Good (nodded). So how many times with that↓
16. Jane: Two.
17. M: YES↓

In this final example of task-focusing (Extract 5), the use of various sequences of utterances, tone and open questioning, provided a setting for an alternating pattern of verbal and non-verbal exchanges during one appointment. These exchanges flowed from triadic, dyadic and back to triadic communication interactions demonstrating the dynamic and cyclical quality of the encounter. This sequence and pattern of communication was particularly evident during task-focusing, when the dental professional was concentrated upon fulfilling the oral health education protocol. Here a dyadic communication interaction (lines 1–7) shifted to a triadic exchange (lines 8–10) as mother intervened to assist John find words to answer the dental professional's question. From this point on (lines 11–15) the exchange reverted to dyadic between John and the dental professional. The interaction returned to triadic as mother intervened again (from line 16 to end). At the close of the encounter the triadic communication pattern returned and was again established, as the task was completed.

**EXTRACT 5:** The dynamic nature of the triadic and dyadic communication interactions.

1. DP: Okay-ducks… (1.0) ((DP put the model back to the counter and sat back on her chair)). So that's how to brush your teeth? How many times a day do you brush your teeth?
2. John: Hmm:::: (1.6) Twi::ce. ((showing 2 with his fingers))
3. DP: ↓TWICE. That's right. Good boy. When do you do it?
4. John: Hmm:::: ((smiling seems thinking hard)) - I don't know ((Jack shrugged his shoulders and then looked at the DP))
5. DP: Do you do it while you (are) lying in your bed sleeping?
6. John: [Uh-huh] ((nodded))
7. DP: NO ((shaking her head))
8. Mother: ((Mum giggled, then child looked at Mummy))
9. DP: (Do) you do it while eating your ↓tea.
10. John: Uh-huh? ((Smiling))
11. DP: NO: ((shaking her head)) (2.0) You don't do that, – you don't sit and eat your ((two arms showing eating behaviour)) – sausages and potatoes when you are brushing your teeth as ↑well:: (at) the same time.
12. DP: When do you clean your teeth?
13. John: I don't know.
14. DP: Yes, you ↓do:::
15. John: No ((smiling and shaking his head))
16. DP: Yes, you do~↓ (nodding head)
17. Mother: Do you do it in the morning, (or) at lunch time - or at bedtime? When do you do it?
18. John: Hmm:::((looking at Mum))– Morning
19. Mother: ((nodded)) And ↑then? What else?
20. John: Uh::: – I don't know, Mum ((looking at his Mum))
21. Mother: At bedtime.
22 John: At bedtime ((turned to gaze at the DP))
23. DP: ↓Excellent.

The ability of the dental professional to maintain symmetry within the triadic communication interaction was affected by the perceived goal or task of the appointment as well as an ability to engage with the parent and provide oral health knowledge in understandable child-centred chunks of information. This type of communication phase was conceptualised as “task-focusing,” associated with achieving Childsmile goals and promoting oral health and therefore predominately reflected the guidance-cooperation phase of Szasz and Hollender (28).

Task-focusing followed a pattern and structure for all dental professionals. While all dental professionals used social talking (dyadic interaction) to welcome parent and child, dentists concentrated upon the dental examination (guidance-cooperation phase) and fluoride varnish application (active-passive phase) before providing any oral health advice, whereas EDDNs spent longer on social talking and containing worries before discussing oral health advice and then applying fluoride varnish (20).

**Figure 3:**
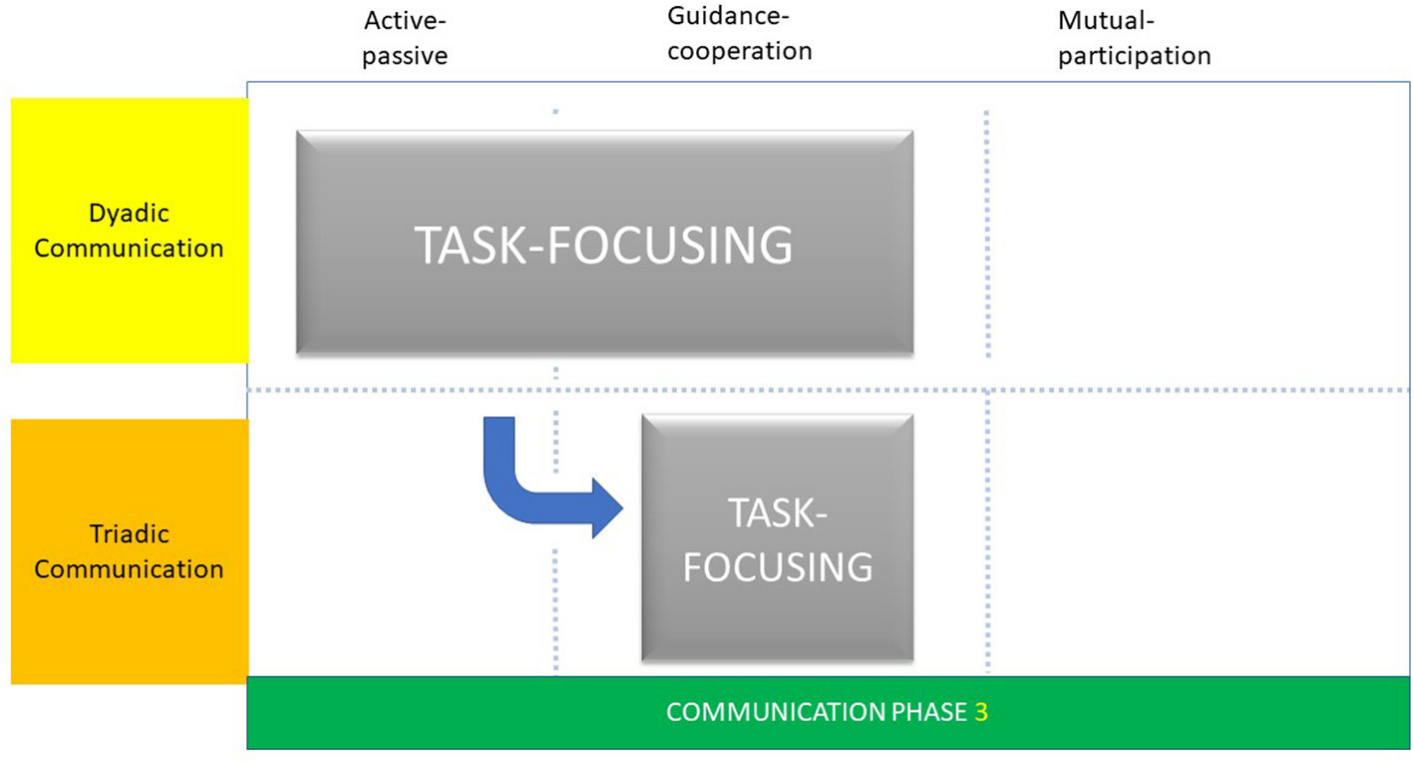
Communication phase 3.

The differences in the patterns and structures of the Childsmile appointment resulted in subtle differences in task-focusing phases. For dentists, task-focusing was characterised by a dyadic communication interaction and symmetrical pattern as described as “adjacent talk-pairs” (Figure 3). This was observed during the Childsmile appointment as the use of a check-list phase of questioning and as short gaps between the questioning by the dentist and answering by the accompanying parent. Irrespective of the age of the child, dentists used this type of interaction which illustrated a form of lexical alignment (35). In this form of lexical alignment, the dentist spoke and listened to the parent simultaneously, and asked questions to shape the parent's responses leading to a pragmatic communication interaction between them. In this respect, the explanatory phase of “guidance-cooperation” was evident as the dentist provided questions (guidance) and the parent complied (cooperation) with appropriate answers.

## Discussion

The aim of the present study was to explore the types of communication interactions and phases that may be exhibited in the paediatric dental encounter, and in particular, those between the dental professional, the parent and the young, preschool child. Taking the opportunity to examine communication during the Childsmile preventive visits enabled us to scrutinise not only the types of communication interactions but the phase of communication adopted.

Using a conversational analytic approach, based on the theoretical perspectives of Finset and Ørnes (25) and Szasz and Hollender's (28) models of clinician-patient interaction, it became possible to suggest that the observational interactions could be characterised as dyadic and/or triadic communication and the specific phases of communication as social talking, task focusing and containing worries. Within each of the dyadic and triadic interactions social talking, task-focusing and containing worries were to a greater or lesser extent used during the communication with parents and children. A careful examination of the empirical data exhibited a dynamic and cyclical quality of the communication interaction. It was not only the character of the interaction that changed during the appointment, but the communication phase was altered to enable the aim of the dental appointment to be achieved. This is reminiscent of Tannen's (19) communication scripts and their use in the various frames of the examination and treatment appointment.

We have described the various communication phases as discrete entities, however, we recognise that the communication phases may cross-over between categories - for example, in Extract 6 – “clean teeth” overlaps between “social talk” and “task focusing.” We propose that the crossing over between communication phases has an equivalence to Tannen's paediatrician's shifts from examination to consultation frames and the accompanying changes in communication. Therefore, while the communication phases are presented as discrete entities, the acknowledgement of their crossing-over during the dental appointment illustrates the dynamic quality of the interaction between dental professional, parent and child and the shifting context of the preventive dental treatment visit.

**EXTRACT 6:** Mike a new patient to the practice.

1. DP: So↑ What do you think? ((DP looked at the child))
2. DP: Come have a seat (DP looked at the Mum and showed her the tub chair with a welcoming gesture))
3. DP: Not you, Mike ↑ ((DP turned to the child when she noticed the child started to climb onto the chair))
4. Mother: Haha…((Laughed and the child jumped away))
5. DP: (2.0) ((DP observed the child's response by gazing at him))
6. DP: Are you happy? ((DP looked at the child when Mum took the seat))
7. Mike: ((Child jumped happily and nodded to the DP))
8. DP: If you're happy, you know what would you do?
9. Mike: ((The child looked at his Mum and Mum looked back to him))
10. DP: If you are happy and you know it, clap your hands…((the DP sang the song and clapped her hands, smiling at the child))
11. Mother: You are shy, you know↑((laughed when gazing at the child as the child did not know how to respond to the DP)). (2.0)
12. DP: So what do you see? (DP gazed at the child with opened arms)
13. Mike: (2.0) Fis ((Child looked around the room))
14. DP: Fish, that's it.
15. Mike: Tus tis
16. DP: What? ((Then the child pointed to the turtle on the wall and looked back to the nurse and Mum))
17. Mother: Turtle? (1.0) right?
18. Mike: ((child nodded and then walked back to the Mum))
19. Mother: Nemo↓ ((Mum pointed to the Nemo figure on the wall)) look
20. Mike: ((Child walked closer to the wall and looked at the wall))
21. Mother: Where is the Nemo?
22. DP: There's some here, too, look ((DP pointed to another wall with Nemo figures))
23. M: Another Nemo ((pointed to the fish on the wall))
24. Mike: ((Child walked toward another wall))
25. DP: What about this one up there? ((pointed at ceiling))
26. Mike /M: ((Both Mum and the child looked up))
27. DP: Look, who's that↓ ((gazed at the child)) (1 sec) Is that Nemo's friend Dory?
28. Mother: (2.0) Yeah↑
29. Mike: This (the) sea horse.
30. DP: It's the sea horse. (1.6) Someone said there might be sharks
31. Mother:.hhh ((Mum had a surprised face)) where's the shark?
32. DP: hiding (1.6) So↓(0.6) Do you know why you are here today?
33. Mike: Clean teeth↑
34. Mother: ((Mum giggled and turned to the nurse as nurse frown)) CLEAN teeth.
35. DP: Clean teeth↓ ((turned to get the prop and then turned back))

Adopting Tannen's idea of “linguistic register” (19) we further propose that differences occurred not only within the duration of the dental visit but also between dentist and the EDDN's interactions between parent and child. For instance, like the paediatrician of Tannen, the dentist in Extract 3 used “an unmarked conversational register” while the EDDNs in their interactions with the child, appeared to use a “teasing register” with “exaggeration in shifts in pitch. and drawn out vowels” as noted in Extract 5 and as coded as “joking and humour” in Yuan et al. (18, 20). Therefore, the different forms of social talking and the ability of the dental professional to alter the content and configuration of social talking illustrates the importance of modifying communication phases or in Tannen's conceptualisation, alterations in communication scripts. For example, at the beginning of the dental appointment, the communication phase social talking assisted relationship-building with the parent and therefore started the process of conversing with the child. This parallels to some extent the important notion coined by the Calgary-Cambridge model of clinical communication where the skills of the clinician assist rapport building through the consultation (36). Consequently, when the child and parent were unknown to the practise and attending for the first time, the role of social talking was of a welcoming format, whereas when the child and parent were known to the practice the social talking was used to welcome but quickly moved to information gathering. Both of these forms of social talking were associated with, especially for the EDDNs, a progression to containing worries and eventually task-focusing.

A careful exploration of these phases of communication, suggested that to a greater or lesser extent the “actors” exhibited repetitive interactive behaviours associated with symmetrical communication patterns or “turn-taking” as conceptualised by Finset A, Ørnes (25) as “adjacent talk-turn pairs.” Adjacent turn-taking was observed when two of the “actors” were talking, with one responding appropriately to the utterances of other. This was observed, for example during social talking and throughout the questioning and answering of task-focusing. Closely associated with adjacent turns, was “lexical alignment.” Thought to ensure effective communication between participants (35), lexical alignment was observed when speakers used similar verbal intonations, pronunciations and even the same words during their conversation. “Lexical repetitions in adjacent turns” (25) were noted, to some degree, in all of the communication phases explored here.

These observations of communication are reminiscent of the phases of health professional-patient interaction as proposed by Szasz and Hollender in their formative paper on the “basic models of the clinician-patient interaction” (28). Proposing a three-fundamental process model of communication, they suggested that interactions could range over three phases “active-passive” to “guidance-cooperation” to “mutual-participation” during one clinical encounter. These phases may not always be in the same order. Likewise, some phases may be absent in some consultations but not in others. This explanatory model may provide a means to understand the subtle changes, observed during the shift in communication interaction during the Childsmile dental appointment. Moreover, it may be suggested that the content of the dyadic communication interaction (for example, task focusing) was suggestive of the active-passive and guidance-cooperation phases whereas the triadic communication interaction (for example, social talking) reflected the guidance-cooperation and mutual participation phases of Szasz and Hollender (28). Dental professionals who adopted these communication phases had the ability to form dyadic and triadic communication interactions with child and parent.

**Figure 4:**
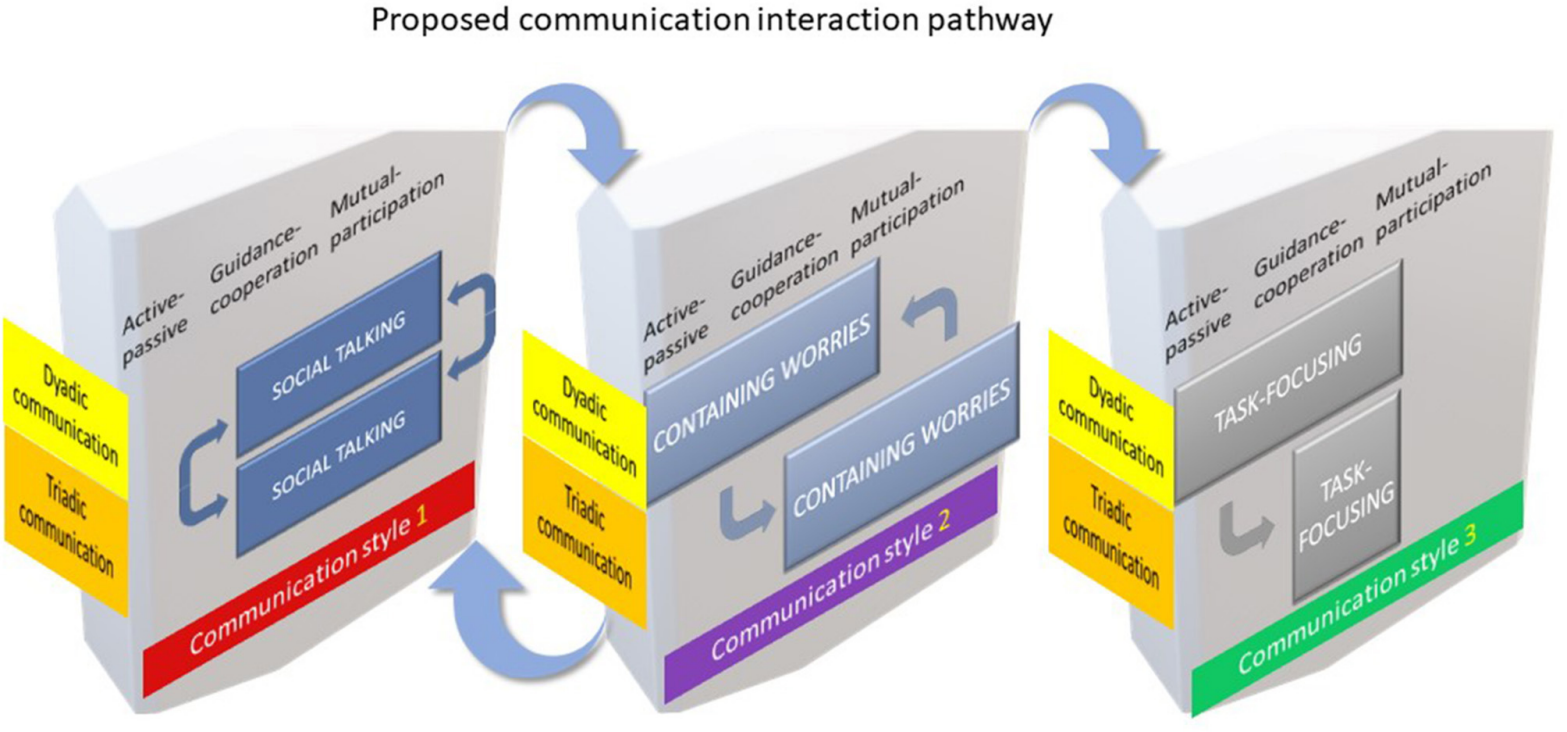
Combined communication pathway model.

We postulate, therefore, that successful social talking heralded the entrance to containing worries and the formation of the triadic treatment alliance. Together social talking and containing worries triggered an integral pathway to task-focusing and achieving the preventive goals of the Childsmile appointment. Therefore, on overviewing the results, we constructed a model to summarise the sequence, relative timing and possible repeated cycling of the patterns of communication phase switching between the dyadic and triadic communication interaction, and across the three phases of Szasz and Hollender (28) clinician – patient relationship model (Figure 4). The three communication phases, namely: social talking, containing worries and task-focusing are displayed as three separate panels in a proposed sequential order in three easily identifiable stages. Each panel represents the Szasz and Hollender framework (28) against the dyadic and triadic communicative behaviours of the “actors” involved. We observed that the first two phases: social talking and containing worries could cycle that is, go back and forth prior to progressing onto the third and final stage of task-focusing. The formation of the treatment alliance, we therefore propose, is essential to enabling task-focusing to proceed. All features of Szasz and Hollender's framework are present in the communication phase, containing worries whereas, only the active-passive and guidance-cooperation elements are apparent in task-focusing. Consequently, the model is not exhaustive nor reflective of every instance but provides a hypothetical overview of the communication interactions and phases adopted by these practitioners.

There is a paucity of research exploring the extent of young children's understanding of oral health information, although another investigation had suggested that 8 to 9-year-old school children have the capacity to assimilate oral health knowledge (37). This present exploratory study indicates that if young children are to comprehend oral health messages, the dental professional must be aware of the parents' health literacy and their health learning capacity (14). Therefore, we propose it is important to acknowledge that the young child's capacity to understand any oral health information on toothbrushing with fluoride toothpaste and/or healthier eating is dependent on dental professionals using words and phrases that are understandable and appropriate to the parent to translate to their child. Using appropriate language and providing limited options, we suggest, enables the parent and then the child to respond appropriately to any dentally-related question. Consequently, the importance of such theoretical perspectives as adjacent turn-taking, and lexical alignment are vital considerations if successful communication interactions are to be achieved during the paediatric dental consultation.

To our knowledge, this is the first study incorporating an explicit theoretical structure using conversation analysis to explore communication interactions and communication phases used by dental professionals with young children and their parents. We acknowledge the relatively small sample size, however, within the paediatric dental appointments videoed, we observed over 7,000 verbal turns and non-verbal cues that permitted close examination of communication between the “actors” who participated. Therefore, while questions may be raised regarding the generalisability of the study findings, we propose that our exploration of the communication interactions and communication phases apparent in the primary dental care setting, permits future work to be focused and to generate additional data to support the hypotheses created here.

In conclusion, the findings of this exploration of the transcriptions of the video data, suggests that the dyadic and triadic communication interactions are of a dynamic and cyclical quality that are exhibited during the paediatric dental consultation. Within each of the dyadic and triadic interactions the communication phases of social talking, containing worries and task-focusing were to a greater or lesser extent used during communication with parents and children. Successful social talking, we propose, signals the entry to containing worries and the formation of the triadic treatment alliance. Future work should generate additional data to support the hypotheses created here.

## Data Availability Statement

The dataset generated and analysed in this article is not publicly available as this was a requirement of ethical approval.

## Ethics Statement

The studies involving human participants were reviewed and approved by East of Scotland Research Ethics Service (Ref: 16/ES/0081). Written informed consent to participate in this study was provided by the participants' legal guardian/next of kin.

## Author Contributions

SY, GH, and RF: methodology. SY: conversational analysis and coding. SY and RF: qualitative analysis and with assistance as required by GH. SY: writing and initial draft. GH: visualization. RF: funding acquisition. All authors writing, reviewing, and editing and conceptualization.

## Conflict of Interest

The authors declare that the research was conducted in the absence of any commercial or financial relationships that could be construed as a potential conflict of interest.
